# Work-Related Musculoskeletal Disorders and Their Associated Factors in Nurses: A Cross-Sectional Study in Iran

**DOI:** 10.21315/mjms2019.26.2.13

**Published:** 2019-04-30

**Authors:** Mohammad Heidari, Mansureh Ghodusi Borujeni, Parvin Rezaei, Shokouh Kabirian Abyaneh

**Affiliations:** 1 Community-Oriented Nursing Midwifery Research Center, Shahrekord University of Medical Sciences, Shahrekord, Iran; 2 Young Researchers and Elite Club, Abadeh Branch, Islamic Azad University, Abadeh, Iran; 3 Shariati Hospital, Tehran University of Medical Sciences, Tehran, Iran; 4 Vice-Chancellor in Treatment Affairs, Shahid Beheshti University of Medical Sciences, Tehran, Iran

**Keywords:** nurse, work-related musculoskeletal disorders, health care workers

## Abstract

**Background:**

Work-related musculoskeletal disorders (WMSDs) in nursing are regarded as an important factor for creating tension since they may often cause discontent, leave profession, and provide incorrect services to their clients. The present study aimed to determine WMSDs and their related factors among the nursing staff in university hospitals affiliated to Shiraz University of Medical Sciences (SUMS).

**Methods:**

In the present descriptive cross-sectional study, 300 nurses in SUMS were selected based on systematic random sampling. To this aim, demographic information, and Nordic musculoskeletal disorder questionnaires were used for data collection. The data were analysed by descriptive and analytical tests (mean, standard deviation, independent *t*-test, and ANOVA) by SPSS/21 software.

**Results:**

Based on the findings of WMSDs, low back disorders (88.33%) were more prevalent. In addition, a significant relationship was observed between WMSDs in different areas of the body with age, sex, and work experience and hours (*P* < 0.05).

**Conclusion:**

Regarding the high prevalence of WMSDs among nurses, it is recommended to adopt interventional program for preventing WMSDs by reducing working hours and physical pressure control.

## Introduction

Jobs play an essential role in social and economic development of any society. Working conditions can lead to numerous problems such as work-related physical disorders which reduce working efficiency (1). In addition, providing many services during the day is considered as one of the requirements for the 24-h community leading to some problems with the workforce due to its overwhelming nature. In this regard, skeletal and muscular disorders are regarded as one of these disorders (2). According to the World Health Organization (WHO), work-related musculoskeletal disorders (WMSDs) lead to their exacerbation when the working activities and conditions are considerably expanded (3). According to National Institute for Occupational Safety and Health (NIOSH), WMSDs are classified as the second most common WMSDs, after occupational respiratory diseases (4). These disorders account for nearly 48% of all diseases (5). Such disturbances may occur gradually during a long process due to long-term exposure to the agents causing these disturbances, or suddenly resulting from a high impact on a part of the musculoskeletal system.

WMSDs are injuries and disorders which affect the human body’s movement or musculoskeletal system (i.e. muscles, tendons, ligaments, nerves, discs, blood vessels, etc.). These injuries are a multi-agent phenomenon. In general, all risk factors are classified into genetic, morphological, psychosocial, and biomechanical factors. Genetic and morphological factors are regarded as non-manipulative, while psychosocial and biomechanical factors are considered as preventable factors in order to prevent injury (6, 7). Physical injuries are related to the gradual damage to the tissues and organs in the body (8). In addition, WMSDs affect the organs of the body involved in the work. Upper organs such as spinal vertebrae and hands are the most sensitive organs against the risk of WMSDs (9).

The WMSDs, as one of the substantial occupational problems, are considered as the most common reasons for losing work-time, and increasing labor costs and injuries (10). Further, these disorders are one of the biggest challenges in occupational health in industrialised countries and one of the most important issues that ergonomists are facing with around the world. In fact, it is regarded as the main concern for public health, leading to temporary or permanent disability among the individuals (11).

Some studies indicated that the WMSDs are considered as the main reason for more than half of workplace absenteeism. Furthermore, some demonstrated that the prevalence and location of pain, and other symptoms may be influenced by body posture and work habits, as well as other demographic factors which are often presented as stiffness, cramps, and muscle soreness with the highest frequency in the neck, shoulders, and lower back (12). Among various occupational groups, health sector professionals, especially those working in the hospital environment, may experience these disorders more frequently. In this regard, nursing is considered as a high-risk occupation for developing diseases (13–15). In addition, it is a profession in which WMSDs are highly prevalent, due to its nature (16).

Nurses are regarded as the fundamental parts of health sector in which some factors such as inappropriate posture during occupational activities, repeated body movements, and long-term and constant pressure to muscles, and the like are responsible for developing these disorders among the nurses (17, 18). Based on the job situation, nurses are responsible for important duties such as psychological and physical care, which necessitate long-term flexion (19). In this regard, flexion is related to those activities, which are performed for transporting the patients, involving the patient’s movement or support such as carrying, pressing, pulling, lifting, and lumbar movements. Compared to carrying objects, carrying a patient or a person is complicated and unpredictable due to his weight and poor grip. Further, he may be scrambled during transport, leading to a lot of damages to the nurse (20, 21).

This class of society is regarded as one of the major contributors in the continuum of care. In fact, healthy nurses are one of the crucial components for improving the care quality (22). In other words, a positive correlation was observed between healthy nursing workforce and health promotion. Regarding physical activity, nursing is in the second rank after industrial occupations (23). In another study in Netherlands, Meijsen and Knibbe reported that the quarterly prevalence of WMSDs was higher among health care workers, compared to that of the general population, which can be evaluated with respect to the prevalence of stress in other stressful occupations, and industry and construction professions (24). WMSDs are considered as an important tension factor among nurses since they may result in creating discontent, quitting profession, and delivering services improperly among the patients (25).

Since WHO is responsible for the health of the society and offering better health services, it should have healthy workforce to achieve this purpose (26). In addition, since WMSDs are economically costly, they are ranked first in terms of pain and suffering which affects the individuals (27). Further, nurses have irregular shifts in the morning, evening and night, along with irregular sleep patterns, and awakening them reduces sleep time and labor power (28). On the other hand, lack of adequate knowledge about standard working conditions, incorrect use of muscles in different situations, as well as lack of physical training, which play a preventive and corrective role, can have a negative effect on the musculoskeletal structure of the body among the nurses, leading to their limb defects (29). Therefore, due to the significance of preventing WMSDs and their consequences in nurses, the present study was conducted to evaluate WMSDs and their related factors among the nursing staff in university hospitals affiliated with Shiraz University of Medical Sciences (SUMS).

## Materials and Methods

### Study Design

The present descriptive study was conducted in university hospitals affiliated with SUMS. The sample included 300 persons estimated by 95% confidence interval, 80% power, and 5% sampling error. They were randomly selected from 35 different sectors in six university hospitals in each work shift. The subjects working officially, by project, or part-time for at least one year before conducting the study were included, while those with the diseases affecting musculoskeletal system and those suffering musculoskeletal injuries during an accident were excluded. In order to comply with ethical issues, the subjects were assured that the questionnaires will remain confidential and the results will only be disclosed generally. Finally, the researcher read the questions for the participants personally and recorded their responses in the questionnaire without any manipulation.

### Ethical Considerations

Nurses who were interested in participating in the study were included in the study. The written informed consent was gained from all participants and they were assured that their provided information will remain confidential. In order to encourage research units to further cooperate, they were assured that the questionnaires would be completed at the free time of each person and would not interfere with their nursing care. In the organisational dimension, researchers and colleagues, managers and all hospital officials will be informed about the research results and the results of this study should be presented in planning and implementing nursing related issues. The present study was approved by Abadeh Branch, Islamic Azad University Ethical Committee.

### Data Collection

In order to collect the data, a questionnaire including demographic information and occupational WMSDs, which was based on Persian version Nordic standardised questionnaire, was used in the present study. In Nordic Musculoskeletal Questionnaire (NMQ), disorders are classified in nine parts of the body based on Kuorinka et al. (32) criteria in order to evaluate WMSDs. The questionnaire can evaluate the problems in the trunk-related organs (neck, shoulders, upper back, lower back), arms (elbow, wrist, fingers), and legs (hip, knee, ankle and toes) among the nursing staff in the workplace (26). The validity of demographic information questionnaire was confirmed by 10 faculty members at SUMS through content validity method. Ozgoli et al. examined the validity and reliability of Nordic standard questionnaire, which was confirmed with the correlation coefficient of 0.91 (30). Further, translation, localisation, formal evaluation, and its repeatability were done in the study of Mokhtarinia et al. (31). Furthermore, Cronbach alpha was used for measuring the reliability among 30 nurses (*r* = 0.90).

### Data Analysis

The data were analysed through mean, standard deviation, independent *t*-test, and ANOVA by SPSS version 21 (IBM Corp., Armonk, NY, USA) software. The reliability of the instrument was confirmed due to repeated use in similar studies, and consensus of the experts. The validity was evaluated by test-retest method and was approved as a screening tool. Based on the results, the questionnaire was found reliable with a correlation coefficient of 91% (32).

## Results

In this study, 300 nurses were enrolled from different work shifts of various wards of hospitals, among whom 89 (29.66%) were men, while 211 (70.33%) were women. In terms of age distribution, the largest age group ranged between 35–45 years. In addition, the highest and lowest age range of participants was 23 and 50 years, respectively. The majority of nurses (66.34%) had 10–20 years of work experience. Other demographic information is presented in Table 1.

Table 2 indicates the prevalence of WMSDs during the last 12 months among the nurses in different parts of the body. The most prevalent disorders were reported in the back (88.33%), knees (83.33%) and thighs (71%). Based on the results, no significant relationship was found between marital status, second job, working system, location, and education level with WMSDs. Further, the results of chi-square test indicated a significant relationship between female gender regarding the musculoskeletal problems in the neck and shoulders (*P* < 0.05). Furthermore, independent *t*-test revealed a significant correlation between age and WMSDs in the neck, shoulders, and knees (*P* < 0.05). Regarding the age group under 25 years, the prevalence of WMSDs in the neck, shoulders, and knees was reported as 11%, 8.3%, and 19.56%, respectively. However, the above-mentioned disorders happened in 39.1%, 37.4% and 62.4% participants in the age group between 35 and 45. The results of logistic regression test showed a positive correlation between knee and wrist pain with working hours per week (*P* < 0.05). In fact, the perception of pain escalated in the knees and wrists by increasing the work hours per week. About 53.2% of the participants working for more than 12 h a day, and 36.2% working 6 h–12 h a day faced with wrist problems. As shown, the difference is statistically significant (*P* = 0.009). In addition, the correlation among WMSDs was found in the knees. Further, the prevalence of knee pain was 37.3%, 20.2%, and 58.1% among those working 6 h–12 h, less than 6 h, and more than 12 h, respectively. However, no significant correlation was observed between pain in other parts of the body and other demographic variables (*P* > 0.05). Table 3 presents the effective demographic factors in creating WMSDs in different areas of nurses. As displayed in Table 4, the results of logistic regression analysis indicated that an increase in the prevalence of problems in most organs can be observed by increasing work experience. Regarding the problems related to the knee (*P* = 0.01), a significant correlation was reported among neck (*P* = 0.025), shoulder (*P* = 0.001) and femur (*P* = 0.006).

## Discussion

The findings indicated that the pain in the back, knees and thighs was more than that of the other areas. In addition, it is suggested that back pain is the most common (88.33%) musculoskeletal disorder among nurses. Some epidemiologic studies indicated an association between occupational factors and WMSDs. Further, some reported that the prevalence and location of pain, along with other symptoms, can be related to standing posture, work habits, and other demographic characteristics (33).

Regarding the prevalence of WMSDs at the back, the results of the present study is consistent with those of Tinubu et al. on nurses (34). In this regard, Magnago et al. reported 71.5% prevalence of back pain in Brazilian nurses, and Maul et al. obtained the rate of 76% in Switzerland (35, 36). In another study, Mehdipour et al. determined the prevalence of WMSDs in operating room personnel. Based on the results, the prevalence of lumbar and back pain was 72.2%, during the last 12 months (37). The findings of Mohseni et al. and Choobineh et al. are in line with those of the present study since they maintained back pain as the most common complication among nurses (38, 39). Nevertheless, Carugno et al. indicated that the shoulders are the most susceptible part for disorders, in their study on Korean nurses (40). Based on the results of chi-square test, no significant relationship was reported between sex and musculoskeletal problems in each of the body parts (*P* > 0.05).

The relationship between gender and musculoskeletal pain can be related to some differences in workload, biological status, and different body size among women and men, which is consistent with those observed in Akrouf et al. and Janwantanakul et al. studies (41, 42). Similarly, a significant relationship was found between gender and low back pain in the study of Spyropoulos et al. (43). This phenomenon may be related to the fact that women often face more psychological stress, compared to men, while doing the same job can influence various aspects of their health including the risk of developing WMSDs. In the present study, a significant correlation was observed between age and pain in the neck, shoulders and knees, which are congruent with the results of Akrouf et al. and Aminian et al. (41, 44). However, regarding the evaluation of the prevalence of the pain related to WMSDs and its association with psychological and social risk factors among office staff, the study results of Aryaie et al. are inconsistent with those in the present study (45).

Logistic regression analysis revealed that a significant relationship between working experience and pain in the hip, neck, knees, and shoulders (*P* < 0.05). In other words, the rate WMSDs significantly increases by increasing work experience, which is in line with the results of Gorgi et al. (46). In addition, Mohseni et al. and Khosroabadi et al. suggested that the prevalence of these disorders increases be increasing work experience (38, 47). In terms of working hours per day, logistic regression demonstrated that the number of working hours per week is positively correlated with the pain in the knees and wrists (*P* < 0.05), which is in agreement with the findings of Giahi et al. They emphasised that short rest periods during 8 hours of work in bank employees can lead to WMSDs (48).

Although the findings indicated high rates of WMSDs in some parts of bodies among the nurses, the present study had some limitations. Personality traits, psychological, social, and cultural backgrounds, along with individual differences and psychological states were among the uncontrollable variables in this study while replying to the questionnaire. Further, the data were collected by self-report, which may fail to reflect the WMSDs among the nurses.

## Conclusion

Based on the results in the present study, it is necessary to adopt interventional program to prevent WMSDs regarding the prevalence of WMSDs in some parts of the body and its significant association with specific demographic characteristics of the nursing profession. Therefore, all the institutions and organisations seeking to improve quality of care by nurses should design necessary plans to manage physical strains, improve working conditions, and reduce working hours.

## Figures and Tables

**Table 1:** Demographic characteristics of the samples (*n* = 300)

Variable		Frequency *N*%	Variable		Frequency *N*%
Marital status	Single	73(24.33)	The number of working hours per day (h)	6 >	11(3.66)
	Married	223(74.33)		6–12	177(59)
	Divorced	4(1.33)		12 <	112(37.33)
Gender	Female	211(70.33)	Level of Education	Bachelor	274(91.33)
	Male	89(29.66)		Master’s degree or higher	26(8.66)
Age (year)	25 >	33 (11)	Work experience (year)	5 >	68(22.66)
	25–35	71(23.66)		5–10	76(25.33)
	35–45	132 (44)		10–20	104(34.66)
	45 <	64(21.33)		20 <	52(17.33)
Residence	Urban	276(92)	Working conditions	Shift	162(54)
	Village	24 (8)		Fixed	138 (46)
Second jobs	Yes	281(93.66)	University educated	Governmental	183(61)
	No	9(6.33)		Non-government	117 (39)

**Table 2:** WMSDs of nurses in different body regions

Body Area	*N*	%
Neck	165	55
Shoulder	77	25.66
Elbow	49	16.33
Hand and wrist	101	33.66
Back	85	28.33
Knee	250	83.33
Femur	213	71
Lumbar	265	88.33
Leg and ankle	138	46

**Table 3:** Factors affecting the occurrence of WMSDs in different areas of the nurses’ body

Body Area	Demographic variables	*P*-value
Neck	Gender	0.001
	Age	0.038
Shoulder	Gender	0.02
	Age	0.022
Hand and wrist	Number of working hours per day	0.009
Knee	Age	0.034
	Number of working hours per day	0.045

**Table 4:** Frequency distribution of WMSDs in terms of work experience in nurses

Work experience (Year)	Body Area								Leg and ankle *N*%

	Neck *N*%	Shoulder *N*%	Elbow *N*%	Hand and wrist *N*%	Back *N*%	Knee *N*%	Femur *N*%	Lumbar *N*%	
**5 >**	25(36.76)	11(16.17)	10(14.70)	36(52.94)	16(23.52)	58(85.29)	19(27.94)	40(58.82)	38(55.88)
**5–10**	42(55.26)	19 (25)	12(15.78)	23(30.26)	42(55.26)	73/(96.05)	59(77.63)	75(98.68)	20(26.31)
**10–20**	47(45.19)	12(11.53)	19(18.26)	18(17.30)	13(12.5)	67(64.42)	85(81.73)	100(96.15)	39(37.5)
**20 <**	51(98.07)	35(67.30)	8(15.38)	24(46.15)	14(26.92)	52(100)	50(96.15)	50(96.15)	41(78.84)
**Total**	165 (55)	77(25.66)	49(16.33)	101(33.66)	85(28.33)	250(83.33)	213(71)	265(88.33)	138 (46)
***P*-value**	**0.025**	**0.001**	**0.189**	**0.28**	**0.358**	**0.01**	**0.006**	**0.600**	**0.35**
